# Pattern and predictors of immunologic recovery in HIV infected patients

**DOI:** 10.1186/s13104-015-1406-3

**Published:** 2015-09-04

**Authors:** Theophilus B. Kwofie, Michael Owusu, Mohamed Mutocheluh, Samuel Blay Nguah, Joseph Obeng-Baah, Charlotte Hanson, Albert Dompreh

**Affiliations:** Department of Clinical Microbiology, Kwame Nkrumah University of Science and Technology, Kumasi, Ghana; Department of Clinical Microbiology, Komfo Anokye Teaching Hospital, Kumasi, Ghana; Department of Child Health, Komfo Anokye Teaching Hospital, Kumasi, Ghana; Department of Medicine, Komfo Anokye Teaching Hospital, Kumasi, Ghana; Laboratory Department, Kumasi South Hospital, Kumasi, Ghana

**Keywords:** HIV, Children, Antiretroviral therapy

## Abstract

**Background:**

Although anti-retroviral therapy has generally improved the survival of HIV infected patients in many developing countries including Ghana, specific socio-demographic factors could still influence outcome of treatment. This study was designed to identify patient-specific factors that could influence the immune recovery of absolute CD4 count in HIV infected patients.

**Findings:**

Hospital records were extracted from two health facilities in Ghana. The impact of socio-demographic factors type of ART and baseline category of CD4 counts were assessed at six monthly interval using robust linear mixed models.

**Results:**

A total of 214 follow up records were reviewed at Komfo Anokye Teaching Hospital (KATH) and the Kumasi South Hospital (KSH). One hundred (46.7 %) were from KATH and 114 (53.3 %) were from KSH. There was a general increase in the level of CD4 counts with time, however this increase significantly slowed down with subsequent reviews (p < 0.001). On the average the rate of CD4 count recovery slowed down by 43.6 cells/µl for every 6 months of follow up (SE = 7.69; p < 0.001). Similarly the recovery of CD4 counts in subjects with an initial high baseline CD4 counts decreased by 192.6 cells/µl (SD error = 42.3, p value ≤0.001). All other variables were not significantly associated with recovery of CD4 counts.

**Conclusion:**

Our study has demonstrated the well-known phenomenon of CD4 counts increasing after administration of ARTs. CD4 counts increased more rapidly in those with relatively lower initial counts, catching up with those with high CD4 count by 2 years post treatment.

## Background

The introduction of highly active antiretroviral therapy (HAART) has slowed down the disease progression of HIV infected patients in both developed and developing countries [1, 2]. Therapeutic success, however, increasingly depends on thorough understanding of the pathogenesis of HIV disease and familiarity with when and how to use the numerous and more effective drugs available to treat HIV infection.

Decisions regarding initiation and/or changes in anti-retroviral therapy (ART) are guided by monitoring the laboratory parameters of plasma HIV RNA (viral load) and CD4+ T cell count in addition to the patient’s clinical condition [3–5]. Results of these laboratory tests provide clinicians with key information regarding immunologic status of the patient and their risk for disease progression to acquired immunodeficiency syndrome (AIDS). In addition, the demonstrations of the prognostic value of the CD4 cell count and the plasma viral load were of major importance in the development of therapeutic strategies.

Some laboratories in resource-constrained countries, however, cannot afford to perform laboratory-monitoring tests of HIV-viral load in particular. As such the World Health Organization (WHO) has advocated CD4 cell count as the basis for monitoring ART in resources-limited settings [6].

The recovery of CD4 T lymphocytes in treated persons is usually accompanied by enhanced T-lymphocyte responses to antigens and mitogens, providing adequate protection against opportunistic infections. It’s utility, however, is influenced by a number of factors including immunological and host specific factors such as age, gender, educational level, socio-economic status etc., [7–9]. The reasons for these different responses remain largely unknown although suggestions have been made that differences in residual thymic function may play a role [10]. Other factors such as the degree of immunodeficiency before initiation of HAART, residual viral activity and viral co-infections such as hepatitis B or C have also been implicated [11, 12]. While the specific factors underlying the long-term recovery of CD4 T lymphocytes deserves particular attention because of its major clinical significance, only few studies have addressed this issue. Information on the socio-demographic and economic factors influencing recovery of CD4 counts among HIV infected patients is limited in many developing countries including Ghana. This study was therefore designed to determine the association of patient-specific factors such as age, gender, socio-economic status and baseline value to four sequential values of absolute CD4 T-cells of HIV-infected patients on ART at the Komfo Anokye Teaching Hospital (KATH) and the Kumasi South Hospital (KSH) in Ghana.

## Methods

### Study site and population

This was a retrospective study carried out between January 2008 and December 2009. The study was carried out at the Serology Units of the Clinical Microbiology laboratory at KATH and the KSH respectively, after appropriate permission has been sought from the respective hospital authorities. The laboratories undertake an external quality assurance system in conjunction with the South Africa Quality Assurance company (AFRQUAS) through the National AIDS Control Programme. KATH is the teaching hospital of the Kwame Nkrumah University of Science and Technology (KNUST) and the only tertiary health institution in the Ashanti region. It is thus the main referral hospital serving predominantly the central and northern sectors of Ghana including the Ashanti, Brong Ahafo, Northern, Upper East and Upper West regions. KSH on the other hand is a relatively smaller hospital which attends to patients mostly from deprived areas of the Ashanti region.

Study populations were children 10 years and above and adult HIV infected patients enrolled on ART programme at the HIV clinic of KATH and the KSH. The first-line treatment available at the two facilities within the period of the study were two nucleoside reverse transcriptase inhibitor drugs (NRTI) being azidovudine (d4T) and lamivudine (3TC) plus a non-nucleoside reverse transcriptase inhibitor (NNRTI) drugs being nevirapine (NVP) or efavirenz (EFV). Protease inhibitor drugs such as Nelfinavir (NFV), Kaletra or Indinavir were also used in combination with the nucleoside based reverse transcriptase inhibitor drugs. Absolute CD4+ T-lymphocyte were quantified using the FACSCount (BD, USA), according to the manufacturer’s instructions.

### Data collection

Patients’ data were collected by reviewing their hospital records for information such as age, sex, marital status, educational level, economic status, religion, availability of family support. Their HIV results and type were noted as well as their CD4 count at both pre- and post-therapy. CD4 counts were recorded before initiation of HAART and at four time points (6, 12, 18 and 24 months) after initiation of HAART. We used a bound of plus or minus 1 week for data collection at the time points. We also excluded children less than 10 years because their data were not readily available as at the time of conducting this research. Data were double entered in spreadsheet database prepared with Microsoft^®^ Excel. It was then compared and cleaned for abnormal wrongful entries.

### Ethical approval

The protocol for this study was approved by the Committee for Human Research, Publication and Ethics of the Komfo Anokye Teaching Hospital (KATH) and the School of Medical Sciences, KNUST, Kumasi, Ashanti region, Ghana. Permission was also sought from the study sites before the data was collected.

### Statistical analysis

Data analysis was performed using R statistical software version 3.0.2 [13]. Continuous variables were expressed as medians with their inter-quartile ranges (IQR) and categorical variables were expressed as percentages.

For the purpose of analysis, economic status of subjects was categorised based on their annual income into low income earners (≤$800), middle income (>$800–US$2000) earners and high income earners (>$2000). These classifications were made based on the occupation of subjects and the US dollar exchanged rates existing in Ghana between the years January 2008 and December 2009. Similarly, the ages of subjects were classified into 10–30, 31–40 and 41–72 years to allow for equivalent distribution.

To determine the overall trajectory of absolute compared to the baseline counts, we categorized the baseline CD4 counts into low and high. Subjects with baseline CD4 counts below 250 cells/µl were categorized as low and those with counts above 250 were categorized high. The 250 cut off value was based on the national treatment policy adopted by Ghana (2004).

The effects of socio-demographic and economic factors on the recovery of CD4 counts were assessed using linear mixed models. Linear mixed model was used to analyse the data because these models are relatively robust to missing values and assume that values missing at random are only dependent on the observed data and not unobserved data [14]. Before using the mixed models, we first evaluated whether missing values were missing at random. We did this by creating a ‘missing’ indicator variable as the outcome variable and then used Chi-square, Student’s t test and logistic regression to evaluate its association with all socio-demographic variables.

The mixed model was defined using the time point changes in CD4 counts as dependent variable and the socio-demographic and economic variables as fixed effects. Each fixed effect factor was tested independently along with subject specific random effect while controlling for time of follow up. The independent factors associated with CD4 count recovery were determined by using significant variables derived from the univariate analysis along with other variables of interest as fixed effects in a full multivariate model. The modelling was done first using subject specific random effect and then time specific random effect. The two models were compared using analysis of variance and the best model determined by choosing the one with lowest Akaike Information Criterion (AIC). All results of the modelling were expressed as the parameter estimate and their standard error. For all analysis, a two-sided p value of less than 0.05 was considered significant. The NLME [15] and EPICALC [16] packages in R were used for all analysis.

## Results

### Subject characteristics

A total of 214 patients record were reviewed at KATH and the KSH. One hundred (46.7 %) were from KATH and 114 (53.3 %) were from KSH. The median age of all study subjects was 37 years (IQR: 32–43). The number of females (159; 74.3 %, 95 %CI 67.9–80.0) was higher than males and most of them were from the KSH. Primary education was the highest (126; 63.6 %) with majority from the KSH. Sixteen (8.1 %) subjects were illiterates. Most of the subjects were in the low economic range (157; 80.1 %) with KSH having the highest. Most subjects also depended on their relatives as the only means of support. Only 29 subjects (14.7 %) could support themselves. Table 1 describes the characteristics of the study subjects.

**Table 1:** Baseline characteristics of study subjects

	Health institution, n (%)		
	KATH	SGH	Total
Gender Female	67 (67.0 %)	92 (80.7)	159 (74.3)
Age (median, IQR)	38 (33–45)	35 (31–42)	37 (32–43)
Educational levels			
None	16 (19.0)	0 (0.0)	16 (8.1)
Primary	49 (58.3)	77 (67.5)	126 (63.6)
Secondary	11 (13.1)	7 (6.1)	18 (9.1)
Tertiary	8 (9.5)	30 (26.3)	38 (19.2)
Economic status			
Low	71 (86.6)	86 (75.4)	157 (80.1)
Medium	10 (12.2)	22 (19.3)	32 (16.3)
High	1 (1.2)	6 (5.3)	7 (3.6)
Primary caregiver			
Spouse	21 (25)	43 (37.7)	64 (32.3)
Relatives	34 (40.5)	57 (50)	91 (46)
Self	25 (29.8)	4 (3.5)	29 (14.6)
Others	4 (4.8)	10 (8.8)	14 (7.1)
Drug			
Azidovudine/lamivudine + NNRTI	39 (50.6)	21 (18.4)	60 (31.4)
Azidovudine/lamivudine + PI	0 (0)	18 (15.8)	18 (9.4)
Stavudine/lamivudine + NNRTI	21 (27.3)	74 (64.9)	95 (49.7)
Others	17 (22.1)	1 (0.9)	18 (9.4)

### Longitudinal changes in absolute CD4 counts

**Fig. 1:**
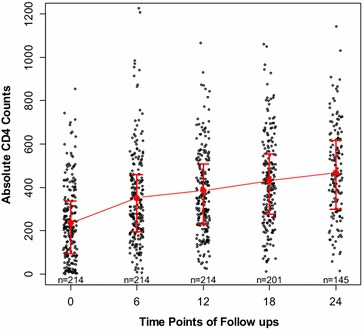
The longitudinal changes of absolute CD4 counts at time points of follow ups. The *red vertical bars* represent the interquartile ranges and the midpoints of the *bars* the medians. The values of “n” depicts the number of subjects who reported to the hospital at the various time points

The median CD4 count before administration of HAART was 212 cells/µl (IQR = 93.5–335.5). The median absolute counts increased significantly (p < 0.001) to 308.5 cells/µl (IQR = 196–457.5) after 6 months and reached a value of 350 cells/µl (IQR = 196–457.5) after 1 year. The median counts reached a peaked value of 453 cells/µl (IQR = 298–616) after 24 months of follow up. Figure 1 describes the medians and interquartile ranges of all CD4 counts at the different time points.

**Fig. 2:**
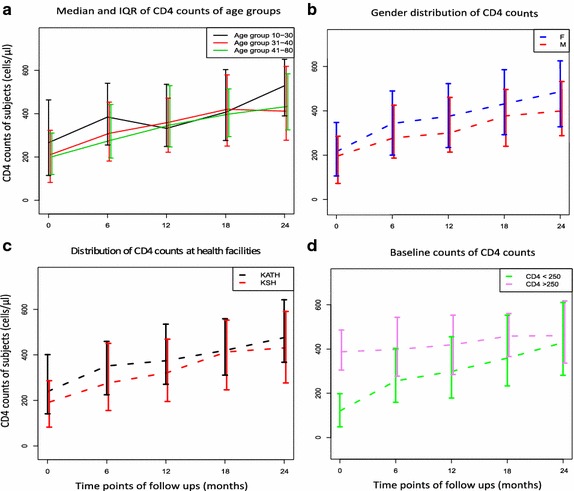
**a** The distribution of absolute CD4 counts for the various age groups. **b** The gender distribution of the changes in absolute CD4 for males (M) and females (F). **c** The distribution of absolute CD4 counts at the study sites. **d** The distribution of absolute CD4 counts for subjects with low and high baseline CD4 counts. The midpoints of the *vertical bars* represent the medians and the heights show the interquartile ranges of the absolute CD4 counts. The x axes show the time points of follow ups

The median CD4 count before administration of HAART for age group 10–30 was 266 cells/µl (IQR = 115–463). This increased to 384 cells/µl (IQR = 255.5–540.0) after 6 months and then decreased to 332 cells/µl (IQR = 248.5–535.5) after 1 year of follow up. Subjects who were in age groups 31–40 and 41–80 years had similar CD4 counts of 210.5 (82.3–323.0) and 201 (120–311) respectively before administration of HAART. After 6 months, the counts increased to 309 (181.25–453.0) and 277.0 (195–442) respectively. These counts increased marginally after 1 year of follow up and then levelled off. Figure 2a describes the median absolute CD4 counts of age groups at the various time points of follow ups.

The median absolute CD4 counts of females were generally higher than males (Fig. 2b). The CD4 counts before administration of HAART were 216 cells/µl (IQR = 106.0–347.5) for females and 197.0 cells/µl (IQR = 72.0–285.5) for males. After 6 months of HAART administration, the counts increased sharply to 343 (200–498.5) and 278 (186.5–425.0) for females and males respectively. These increments continued with females showing higher counts than males.

We also compared the CD4 counts recorded at KATH and the KSH. The median CD4 counts before administration of HAART were 239 cells/µl (IQR = 141.25–401.5) and 192.5 cells/µl (IQR = 83–286) for KATH and KSH respectively. These counts increased steadily to medians of 351.5 cells/µl (IQR = 225–459.3) and 277.0 cells/µl (IQR = 156–451) for KSH and KATH patients respectively after the 6 months of HAART administration. The counts further increased marginally to 374.5 (IQR = 271.3–535) and 322.5 (IQR = 195–469) and then peaked together at 420.0 (IQR = 311–558.5) and 413.5 (247–552.5) respectively for KATH and KSH. Generally, the CD4 counts for KATH subjects were higher than those at KSH although the difference was not significant. Figure 2c describes the CD4 counts of subjects recorded at the two institutions.

The median baseline CD4 counts for low category subjects was 120 (IQR = 48–198) and that for high category subjects was 388 (IQR = 305–486). Twelve months after baseline HAART administration, the CD4 counts for low category subjects increased sharply to a value of 299 (IQR = 178–456) while that of high category subjects only increased marginally to 420 (IQR = 285–553). After 12–18 months of HAART administration, the absolute CD4 counts plateaued for high category subjects but that of low category subjects rose steadily and caught up with values of high category subjects at 24 months. Figure 2d describes the trajectory of absolute CD4 counts for low and high category subjects.

### Predictors of rate of absolute CD4 count recovery

There was a general increase in the level of CD4 count with time, however this increase significantly slowed down with subsequent reviews (p < 0.001). On the average, the rate of absolute CD4 counts recovery slowed down by 28.6 cells/µl for every 6 months of follow up.

Though CD4 counts for persons with low and high baseline counts both rose with time the rate of rise was significantly slower in those with high baseline compared to those with low baseline (difference: 65.7 cell/μl per 6 months, SE = 12.99, p < 0.001). All other variables were not significantly associated with the rate of recovery of CD4 counts. Table 2 describes the predictors of rate of changes in absolute CD4 counts.

**Table 2:** Predictors of rate of CD4 count recovery

Predictor	Parameter estimate	Standard error	P value
Intercept	56.7	6.6	<0.001
Time	−28.6	6.0	<0.001
Age (years)	−0.3	0.7	0.698
Gender			
Female (reference)			
Male	−12.3	14.8	0.410
Primary caregiver			
Parents (reference)			
Relatives	0.15	14.9	0.992
Self	−17.8	20.1	0.379
Other	−0.3	26.6	0.991
Economic status			
Low (ref)			
Medium	6.4	17.6	0.714
High	−6.0	34.1	0.860
Educational levels			
None (reference)			
Primary	−20.4	24.5	0.407
Secondary	−33.1	31.7	0.298
Tertiary	−30.5	27.4	0.267
Place			
KATH (reference)			
Suntreso Gov. Hospital	9.6	13.0	0.459
ART regimen			
Others (ref)			
Azidovudine/lamivudine + NNRTI	4.9	25.0	0.845
Azidovudine/lamivudine + PI	8.1	31.5	0.797
Stavudine/lamivudine + NNRTI	3.8	23.9	0.873
Baseline CD4 level			
Low (≤250/µl) (ref)			
High (>250/µl)	−65.7	13.0	<0.001

In the final multivariate analysis, significant variables such as baseline CD4 counts and time of follow ups were included as an interactive term. The results showed that the average rate of CD4 count recovery slowed down by 43.6 cells/µl for every 6 months of follow up (SE = 7.69; p < 0.001). Similarly the rate of CD4 count recovery for subjects who initiated ARTs with high baseline counts was lower than those who started with low baseline CD4 counts. On the average, the CD4 counts of subjects on initial high baseline CD4 counts decreased by 192.6 cells/µl (SD error = 42.3, p value ≤0.001) for every 6 months of follow up compared to those with low baseline CD4 counts.

## Discussion

This study determined retrospectively the association of patient-specific factors such as age, gender and socio-economic status to the immunological response of HIV-infected patients receiving anti-retroviral therapy at the KATH and KSH. The result of the study showed a sustained immune recovery of CD4 counts over time. The rate of recovery however slowed down with time. The general increase in the absolute CD4 counts corroborate other reports about the efficacy of NRTI and NNRTI-based HAART regimens in HIV-infected patients in resource limited countries [17–19].

This study also compared the absolute CD4 counts for the different age categories. The overall difference was however not significant. Younger age groups have however been reported by other authors to have better immune CD4 count recovery [20, 21].

An evaluation of the association between HIV patient caregiver status and the rate of CD4 count recovery did not yield any significant difference. A recent study among HIV infected children (age 0–13 years), reported by Barry et al. [22], however identified having biologic parents as an independent factor associated with absolute CD4 count recovery. This could possibly mean caregiver status may not play any special role in improving CD4 count recovery in older children and adults. However our study may not have been sufficiently powered to fully evaluate this association.

Our study did not find significant association between gender and rate of recovery of absolute CD4 counts. The relationship between CD4 count recovery and gender has received mixed reviews [23–25]. Sempa et al. [26] reported response higher probability of CD4 recovery in females compared to males [26]. Teshome and Assefa [27] on the other hand did not find significant association in terms of immunological recovery [27]. Our result is consistent with the latter report and further suggests the need for in-depth investigation into this phenomenon.

Our study also revealed greater increases in CD4 counts for subjects with low baseline CD4 counts over the entire duration of the study, compared to subjects with high baseline CD4 counts. Other studies have similarly reported long term significant increases in CD4 cell counts among subjects with low baseline CD4 counts [28, 29].

These findings could be due to the redistribution of sequestered cells T cells after administration of ARTs and the gradual increase of naïve cells because of the diminishing antigenic pressure [30]. One limitations of this study is the few missing values recorded for some of the CD4 count values during follow ups. We also did not investigate the influence of resistant strains of HIV in our analysis.

In conclusion, our study has shown the effectiveness of ARTs in improving the recovery of CD4 counts over time, particularly for subjects who initiate therapy with low baseline absolute CD4. Future studies could explore the role of virological loads and other host genetic factors on the recovery of absolute CD4 counts.

## Abbreviations

3TC: lamivudine
AIDS: acquired immunodeficiency syndrome
ARTs: anti-retroviral drugs
d4T: azidovudine
EFV: efavirenz (EFV)
HAART: highly active antiretroviral therapy
HIV: human immunodeficiency deficiency syndrome
IQR: interquartile range
KATH: Komfo Anokye Teaching Hospital
KNUST: Kwame Nkrumah University of Science and Technology
KSH: Kumasi South Hospital
NNRTI: non-nucleoside reverse transcriptase inhibitor
NVP: nevirapine (NVP)
WHO: World Health Organization
